# Isatin-Schiff base-copper (II) complex induces cell death in p53-positive tumors

**DOI:** 10.1038/s41420-018-0120-z

**Published:** 2018-11-13

**Authors:** Emil Bulatov, Regina Sayarova, Rimma Mingaleeva, Regina Miftakhova, Marina Gomzikova, Yuri Ignatyev, Alexey Petukhov, Pavel Davidovich, Albert Rizvanov, Nickolai A. Barlev

**Affiliations:** 1 Kazan Federal University, Kazan, Russian Federation; 2 Institute of Cytology of Russian Academy of Sciences, St. Petersburg, Russian Federation; 3 St. Petersburg State Institute of Technology, St. Petersburg, Russian Federation; 4 Present Address: Trinity College, Dublin, Ireland

## Abstract

Medicinal bioinorganic chemistry is a thriving field of drug research for cancer treatment. Transition metal complexes coordinated to essential biological scaffolds represent a highly promising class of compounds for design of novel target-specific therapeutics. We report here the biological evaluation of a novel Isatin-Schiff base derivative and its Cu(II) complex in several tumor cell lines by assessing their effects on cellular metabolism, real-time cell proliferation and induction of apoptosis. Further, the impact of compounds on the p53 protein and expression of its target genes, including *MDM2*, *p21/CDKN1A*, and *PUMA* was evaluated. Results obtained in this study provide further evidence in support of our prior data suggesting the p53-mediated mechanism of action for Isatin-Schiff base derivatives and their complexes and also shed light on potential use of these compounds for stimulation of apoptosis in breast cancer cells via activation of the pro-apoptotic *PUMA* gene.

## Introduction

Discovery of metal complexes as antitumor chemotherapeutics, such as cisplatin, led to a dramatic shift of focus toward bioinorganic/organometallic compounds containing transition metals and their chelates as novel scaffolds for drug discovery and development^1–3^. Enormous interest has emerged for the development of various platinum-based analogs since the serendipitous discovery of cisplatin by Barnett Rosenberg in 1960s. Following approval and launch of this drug and its second-generation analogs for the use as chemotherapeutic treatments against testicular and ovarian cancers^4^ a large number of novel metal-based anticancer therapeutics (metallodrugs) have entered into the preclinical development and even clinical trials as antitumor drugs, radiopharmaceuticals, and magnetic resonance imaging contrast agents^5,6^.

Various chemical scaffolds are utilized for design of potent metallodrugs, one of them is a natural product Isatin (1H-indole-2,3-dione) that is found in many plants. Isatin derivatives represent scaffolds that are privileged in medicinal chemistry space and also serve as a common structural motif for a wide variety of pharmaceutically active compounds. Isatin-Schiff base Cu(II) complexes represent synthetic compounds with a broad scope of biological activity, including antimicrobial, antibacterial, and antifungal properties^7–11^. Previous studies demonstrated that Cu(II) complexes of Isatin-Schiff base derivatives (ISBDs) are able to negatively affect tumor cell viability and induce pro-apoptotic activity in neuroblastoma SH-SY5Y^12–14^, colorectal carcinoma HCT116^15^, and melanoma TM1 cell lines^16^.

To reduce potential side effects and maximize efficiency it is important to retain the target specificity of small molecule therapeutics. In the last decade, the ubiquitin-proteasome system (UPS) and its components have emerged as key targets for the development of novel small molecule therapeutics. The drug discovery research that targets E3 ubiquitin ligases, enzymes conferring the substrate specificity to the UPS, gains a lot of interest due to the paramount role of these enzymes in a wide range of biological processes and human diseases, including cancer and autoimmune disorders^17–21^. There are numerous examples of Cu(II) complexes^22–25^ as well as Isatin derivatives^26^ acting as antitumor therapeutics that target various components of UPS, mainly the proteasome and E3 ubiquitin ligases^27,28^. One important example is MDM2 (murine double minute 2), an E3 ubiquitin ligase overexpressed in many tumor cell lines, which functions as a primary negative regulator of tumor suppressor protein p53^29,30^. MDM2 exerts its regulatory function on p53 by polyubiquinating it for subsequent proteasomal degradation^31^. Inhibition of MDM2 by small molecules is considered as one of the major strategies for activation of p53 and its transcriptional functions, further leading to apoptosis and cell cycle arrest^32^.

Here, we report a novel Isatin-Schiff based derivative Cu(II) complex (Complex) that exhibits cytotoxic properties toward p53-positive MCF7 tumor cells, promotes p53-dependent gene expression and induces apoptosis. In support of our previously reported data^33^ we provide evidence for the p53-mediated mechanism of action by quantitative analysis of *MDM2*, *p21/CDKN1A*, and *PUMA* genes expression.

## Results

### Isatin-Schiff base-copper(II) complex activates p53 protein

**Fig. 1:**
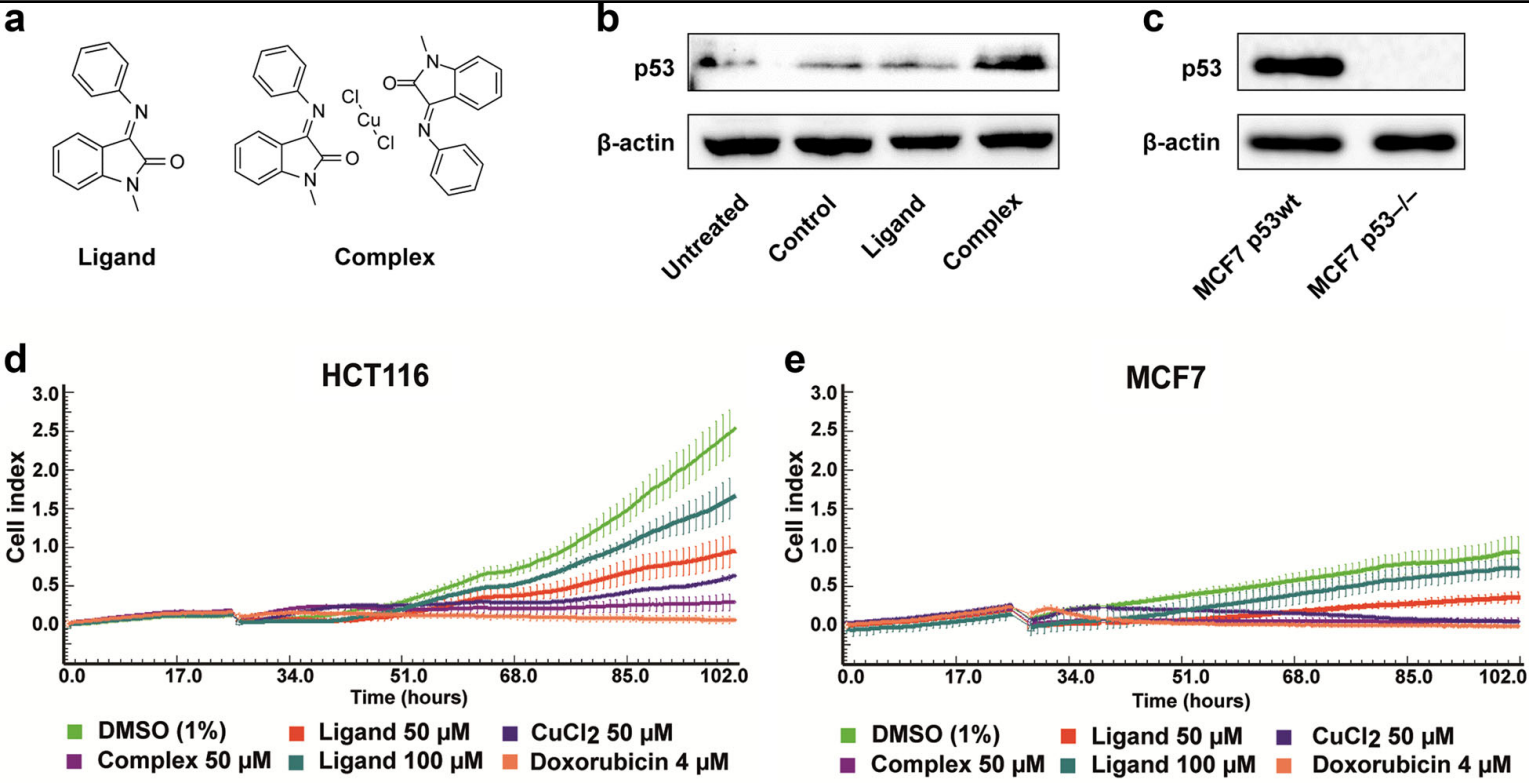
Complex activates p53 and reduces proliferation of HCT116, MCF7 tumor cells. **a** Chemical structures of Isatin-Schiff base and its copper(II) complex. (E)-1-methyl-3-(phenylimino)indolin-2-one and (E)-1-methyl-3-(phenylimino)indolin-2-one copper(II) chloride complex are named as Ligand and Complex, respectively. **b** Complex, but not Ligand, activates p53 protein. Immunoblot analysis of MCF7 p53wt cells treated with Complex (50 µM), Ligand (50 µM), and DMSO (1%, vehicle control) for 24 h revealed Complex-mediated activation of p53 protein. **c** Lack of p53 protein expression in MCF7 p53^−/−^ validated by immunoblotting. MCF7 p53^−/−^ cells were obtained using CRISPR/Cas9 knockout of *TP53* gene in MCF7 p53wt cells. Monoclonal populations of MCF7 cells containing frameshift in double-strand break region of *TP53* gene were analyzed by immunoblotting to confirm lack of p53 protein expression. As a control we used mixed population of MCF7 cells stably transduced with lentivirus encoding pCW-Cas9 and transiently transfected with pLenti-SG1 construct encoding scrambled sgRNA. Both MCF7 p53wt and MCF7 p53^−/−^ cells were incubated with 1.5 µg/ml doxorubicin for 20 h prior collection and lysis. **d**, **e** Isatin-Schiff base-copper(II) complex negatively affects proliferation of HCT116 and MCF7 tumor cells. **d** HCT116 and **e** MCF7 cells were seeded at 5,000 per well in E-Plates 16 and were treated with Ligand (50 and 100 μM), Complex (50 μM), CuCl_2_ (50 μM), and doxorubicin (4 μM) for 72 h. Treatments were performed in triplicates within each plate. Cell index parameter was recorded every 15 min

One of the main objectives of the current study was to explore potential antitumor properties of Complex (Fig. 1a) in p53-positive tumors such as breast cancer MCF7 cell line. As evident from the immunoblot analysis (Fig. 1b), treatment of MCF7 p53wt cells with Complex results in substantial increase of p53 protein levels, at the same time Ligand, the chelating component of Complex, on its own had no effect on p53 activation.

To further validate p53-associated effects of Complex on tumor cells we decided to compare isogenic cell lines that differ in their p53 status (p53 positive vs. p53 negative). To this end, MCF-7 tumor cells were subjected to CRISPR/Cas9 genome editing to obtain homozygous MCF7 p53^−/−^ cell line. Lack of p53 protein in MCF7 p53^−/−^ cells was confirmed by immunoblot analysis after doxorubicin treatment to induce maximum p53 levels upon DNA damage activation (Fig. 1c).

To further investigate cytotoxic effects of the compounds on tumor cells we carried out real-time monitoring of proliferation using the xCELLigence biosensor array system. This method allows noninvasive quantification of cell growth by measuring impedance-based electronic readout (cell index parameter) that correlates with the number of surface-adherent cells. Two cancer cell lines of different origin (HCT116 and MCF7, colon and breast cancer, respectively) with wild-type p53 were treated with Ligand (50 and 100 μM), Complex (50 μM), CuCl_2_ (50 μM), and doxorubicin (4 μM) for 72 h, during this period the cytotoxic response kinetics were monitored in real time. To rule out a possibility that cytotoxicity of Complex could be due to intracellular disintegration of the metal complex into its primary components, Isatin-Schiff base and Cu(II) ions, we assessed cytotoxicity of Complex and Ligand separately. As demonstrated in Fig. 3, the characteristic kinetic profiles indicate that Complex and CuCl_2_, likewise doxorubicin, substantially attenuated proliferation of both cell lines compared to control sample (1% DMSO). For both HCT116 and MCF7 cell lines Complex inflicted substantially higher than Ligand cytotoxicity comparable with doxorubicin treatment (Fig. 1d, e).

We also noticed that in general, MCF7 cells were more sensitive to the treatment with Complex in comparison with HCT116 cells (compare Fig. 1e, d, respectively). Therefore, we decided to conduct our subsequent experiments using both MCF7 p53wt and MCF7 p53^−/−^ cells to examine Complex-mediated impact on cell viability, proliferation, apoptosis, and expression of p53 target genes.

### Isatin-Schiff base-copper(II) complex negatively affects proliferation of p53-positive MCF7 tumor cells

**Fig. 2:**
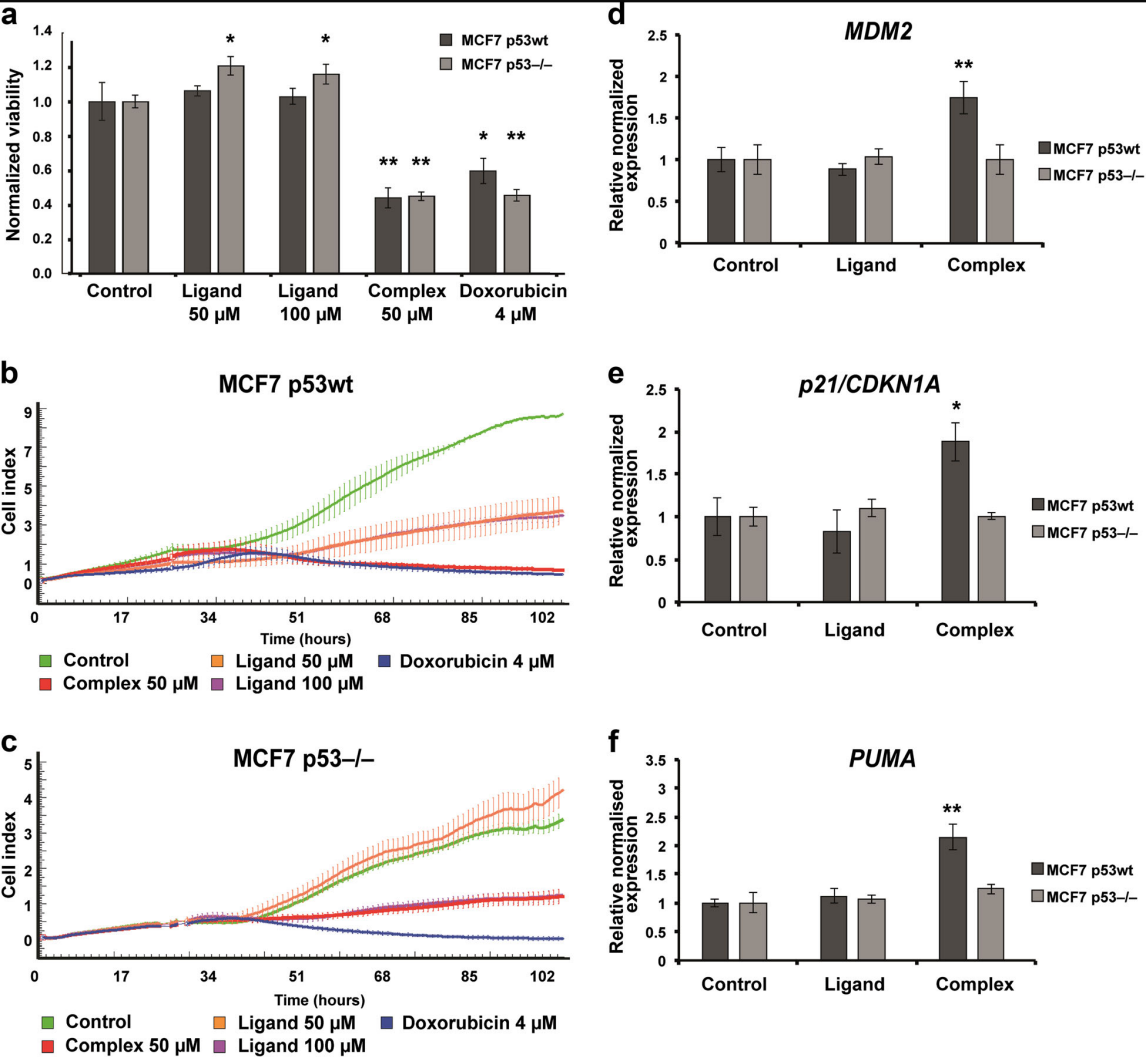
Complex reduces proliferation of MCF7 cell line and enhances expression of p53 target genes in p53-dependent manner. **a** Complex negatively affects viability of MCF7 p53wt and MCF7 p53^–/–^ tumor cells. MCF7 p53wt (dark gray), MCF7 p53^−/−^ (light gray) cells were treated with Complex (50 μM), Ligand (50 μM and 100 μM), DMSO (1%, vehicle control), and doxorubicin (4 μM) for 24 h. Cell viability was evaluated by colorimetric MTS assay. Ligand demonstrated no cytotoxicity for either of the cell lines at both concentrations, whereas Complex substantially reduced viability that was comparable to doxorubicin at indicated concentration. Data are expressed as mean ± S.D., *n* = 3; ^*^p < 0.05, ^**^p < 0.001. **b**, **c** Complex blocks proliferation of MCF7 p53wt but not MCF7 p53^−/−^ tumor cells. **b** MCF7 p53wt and **c** MCF7 p53^−/−^ cells were seeded at 5 × 10^3^ per well in E-Plates 16 and treated with Complex (50 μM), Ligand (50 μM and 100 μM), DMSO (1%, vehicle control), and doxorubicin (4 μM) for 72 h. Treatments were performed in triplicates within each plate. Cell index parameter was recorded every 15 min. **d**–**f** Complex promotes expression of p53 target genes in MCF7 p53wt but not in MCF7 p53^−/−^ cells. Relative normalized expression levels of *MDM2*, *p21/CDKN1A*, and *PUMA* genes in MCF7 p53wt and MCF7 p53^−/−^ cells after 24 h treatment with Complex (50 μM), Ligand (50 μM), and DMSO (1%, vehicle control). Data are expressed as mean ± S.D., *n* = 3; ^*^p < 0.05, ^**^p < 0.001

Initial cytotoxicity experiments were carried out using colorimetric MTS assay to determine viability of MCF7 (p53wt and p53^−/−^) tumor cell lines following 24 h treatment with the compounds (50 µM Complex, 50 µM and 100 µM Ligand). Ligand demonstrated no cytotoxicity for either of the cell lines, whereas treatment with Complex substantially reduced viability of both cell lines (Fig. 2a). Results are consistent with the previously reported observations suggesting that metal complexes demonstrate higher cytotoxicity and more dramatic reduction of proliferation of tumor cells compared to free ligands^34^.

To investigate whether the cytotoxic effect of Complex on tumor cell proliferation was p53-dependent we carried out real-time monitoring of cell division using the xCELLigence biosensor array. MCF7 p53wt and MCF7 p53^−/−^ cells were treated with Complex (50 μM), Ligand (50 μM and 100 μM), and doxorubicin (4 μM) for 72 h, during this period the cytotoxic response kinetics was monitored in real time.

As demonstrated in Fig. 2b, c the characteristic kinetic profiles indicate that although Complex substantially attenuated the proliferation of both p53-positive and p53-negative MCF7 cells compared to control (1% DMSO), the effect was more dramatic in case of p53-positive cells (compare Fig. 2b, c). On the contrary, we observed a significantly lower Complex-mediated cytotoxic response in MCF7 p53^−/−^ cells (Fig. 2c), i.e., cells continued to proliferate, although at a much slower rate. Altogether, these data point at the likely p53-mediated mechanism of cytotoxicity.

### Isatin-Schiff base-copper(II) complex enhances gene expression in p53-dependent manner

Transcription factor p53 plays a central role in triggering cell cycle arrest and apoptosis via activation of both coding and noncoding genes^35,36^. Our previously developed ISBDs demonstrated p53-activating cellular effect, presumably occurring via the MDM2 inhibition. Therefore, we expected that Complex would act similarly. To evaluate the effect of Complex on the expression of p53 target genes we used Taqman real-time reverse transcription quantitative polymerase chain reaction (RT-qPCR). We observed that Complex promoted expression of the key p53-regulated genes including *MDM2*, *p21/CDKN1A*, and *PUMA* (Fig. 2d–f). In contrast, no alteration of gene expression was observed in MCF7 p53^−/−^ cells treated with Complex, further suggesting that the latter operates via the p53-associated mechanism. Importantly, since *PUMA* is a crucial mediator of both p53-dependent and p53-independent apoptosis^37^, we decided to further evaluate the effect of Complex on apoptosis.

### Isatin-Schiff base-copper(II) complex induces apoptosis in both MCF7 p53wt and MCF7 p53^−/−^ cells

**Fig. 3:**
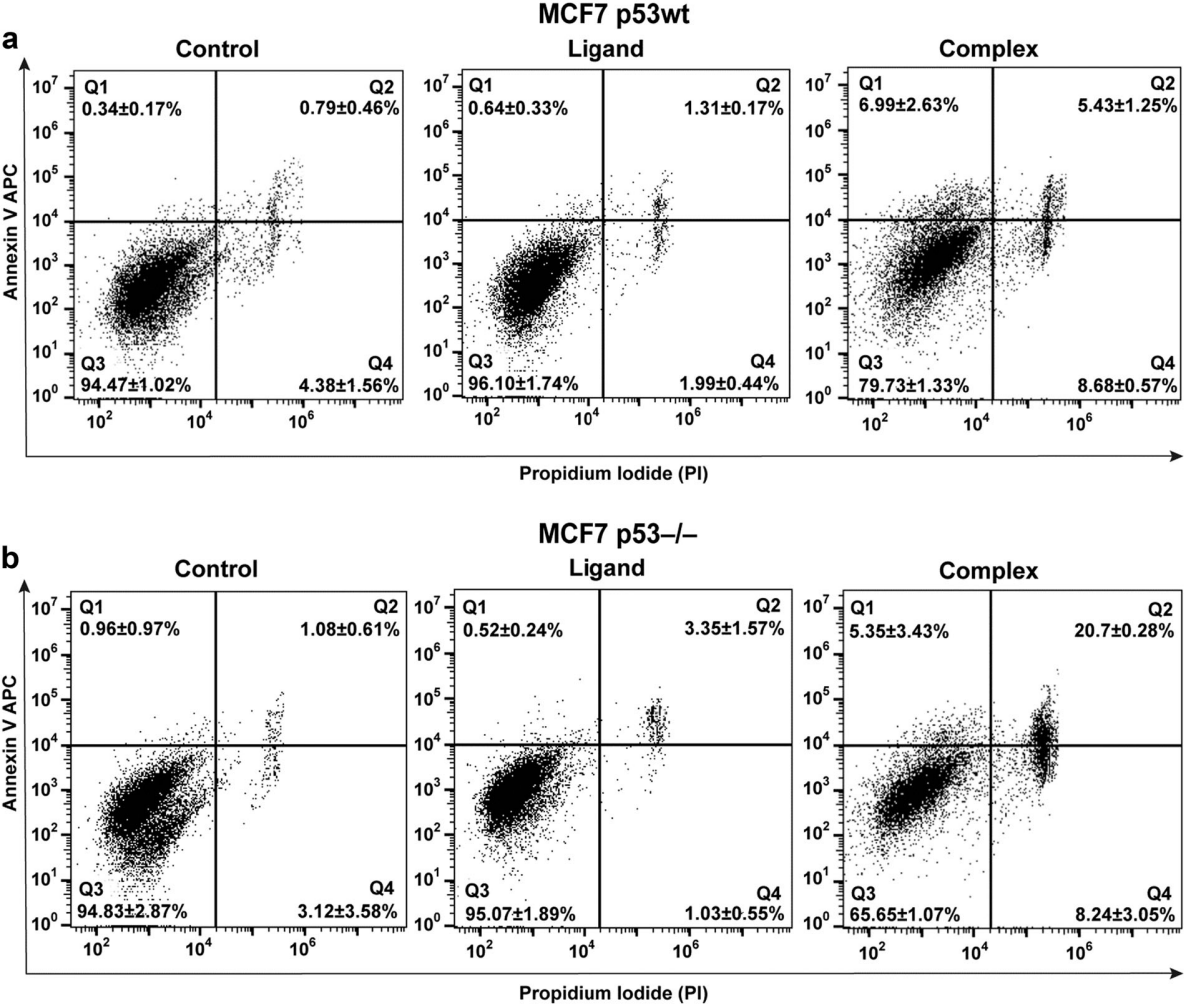
Complex induces apoptosis and necrosis in MCF7 p53wt and MCF7 p53^–/–^ tumor cells. **a** MCF7 p53wt and **b** MCF7 p53^−/−^ cells were treated with Complex (50 μM), Ligand (50 μM), and DMSO (1%, vehicle control) for 48 h, then stained with APC Annexin V and propidium iodide to evaluate percentage of apoptotic and necrotic cells. Stained cells were analyzed by flow cytometry using BD FACSAria III and data processed with FlowJo software package. Quadrants represent following: Q1—early apoptotic cells (Annexin V positive); Q2—late apoptotic and necrotic cells (double positive); Q3—healthy live cells (double negative); Q4—necrotic cells (PI positive). Diagrams show one representative dataset out of three independent experiments. Percentages are expressed as mean ± S.D., *n* = 3

To determine whether Complex decreased cell survival by induction of apoptosis MCF7 cells and their p53^−/−^ progeny were treated with Complex (50 μM), Ligand (50 μM), and DMSO (1%, vehicle control) for 48 h and then stained with APC Annexin V and Propidium Iodide to evaluate percentage of apoptotic cells by flow cytometry. Results obtained indicate a significant increase in the percentage of late apoptotic and necrotic cells upon treatment with Complex of both MCF7 p53wt and MCF7 p53^−/−^ cells (Quadrants Q1, Q2 in Fig. 3a, b). At the same time, Ligand did not exhibit any pro-apoptotic activity in neither of the cell lines. Rather unexpectedly, we observed higher percentage of late apoptotic cells in Complex-treated p53-negative cells, compared to the p53-positive ones.

Collectively, our results suggest that Complex affects proliferation of cancer cells in both p53-dependent and independent manner. However, the presence of p53 exacerbates the cytotoxic effect of Complex.

## Discussion

Currently, design and synthesis of highly potent metallodrugs is topical area of research with numerous enzymes, transcription factors, and major pathway components as molecular targets^38^. Transition metals offer considerable advantages for drug development such as ability to form geometrically diverse coordination complexes with metal ions in different oxidation states. The prominent examples in this field, cisplatin, still remains one of the most widely used antitumor drugs in clinics. However, despite all the benefits and general clinical success there are major disadvantages associated with cisplatin-based drugs such as severe side-effects, toxicity, low solubility, and acquired resistance in certain types of tumors^39^. Attempts to overcome these limitations inevitably led to broad efforts toward development of diverse antitumor agents based on transition metal complexes such as ruthenium, copper, cobalt, palladium, titanium, gold, and other^40–42^.

Our broad research interest lays in determining how transition metal complexes, like Cu(II), could improve and enhance antitumor therapeutic properties of Isatin core and its Schiff base derivatives. The choice of Schiff base system was due to several reasons: chemical scaffold accessibility, its widely known role in coordination chemistry, and the ability to stabilize metal ions in various oxidation states. We have previously identified small molecule ISBDs that demonstrated p53 activation in U2OS osteosarcoma cells^33^. Primary in silico screening and subsequent docking experiments performed for p53-binding pocket of MDM2 yielded ISBDs acting as p53 α-helix mimetic. These compounds are represented by two isomers (*E* and *Z*) that were shown to exist in dynamic equilibrium in solution and could transform via the intramolecular isomeric interconversion. The recent data demonstrate that this conformational interconversion could be ceased by methyl substitution of the Isatin aromatic moiety alongside coordination with transition metal ions, such as Cd(II) and Hg(II)^43^. Subsequently, ISBD Cu(II) complexes stabilized by both metal ion coordination and methyl substitution of the Isatin core have been designed and synthesized to specifically induce p53.

In the current study, we investigated the effect of Complex on p53-positive tumor cell viability, proliferation and apoptosis. We also confirmed Complex-mediated stabilization of the p53 protein and subsequent transcriptional activation of primary p53 target genes such as *MDM2, p21/CDKN1A*, and *PUMA*^44^.

The PUMA protein is well-known for its regulatory role in programmed cell death. Therefore, activation of *PUMA* is considered as a promising therapeutic strategy to inhibit tumor growth by restoring apoptosis in cancer cells ^37^. Complex-promoted *PUMA* expression in MCF7 p53wt cells (Fig. 2f) provides evidence in support of p53-mediated mechanism of pro-apoptotic activity. However, a more detailed analysis of apoptosis revealed that Complex induced programmed cell death in both MCF7 p53wt and p53^−/−^ cells, suggesting existence of an additional p53-independent mechanisms of cytotoxicity. Given this, one might speculate that Isatin-Schiff base-copper (II) complex has a dual mechanism of action—through direct inhibition of MDM2, as previously reported^33^, and also via eliciting oxidative stress or DNA damage, similar to some other metal complexes ^45^.

Conclusions that can be drawn from our results are fourfold: (i) Complex exhibits substantial cytotoxicity toward p53-positive and p53-negative MCF7 cells according to cell viability assay; (ii) Complex blocks preferentially proliferation of the p53-positive but not p53-negative MCF7 cells, as determined by real-time cell proliferation analysis; (iii) Complex promotes expression of p53-target genes *MDM2*, *p21/CDKN1A*, and *PUMA* in p53-dependent manner; (iv) Complex induces apoptosis in both p53-positive and p53-negative MCF7 cells.

Future studies in the area of developing Isatin-Schiff ligands coordinated with various transition metal ions are expected to yield scaffolds based on Isatin-Schiff metal ion complexes with enhanced potency and target specificity. Such compounds could provide an alternative to platinum-based antitumor drugs and potentially help overcome the resistance toward cisplatin chemotherapy.

## Materials and methods

### Materials

(E)-1-methyl-3-(phenylimino)indolin-2-one (Ligand) and (E)-1-methyl-3-(phenylimino)indolin-2-one copper(II) chloride complex (Complex) were synthesized and characterized. Dimethyl sulfoxide was from Sigma-Aldrich. Primers and Taqman probes were from Lytech (Russia). Antibodies used were anti-p53 antibody [DO-1] (ab1101, Abcam), anti-mouse IgG–Peroxidase antibody (A4416, Sigma Aldrich), THE Beta Actin antibody [HRP] (A00730, GenScript).

### CRISPR/Cas9 knockout

Heterozygous MCF7 p53^−/−^ cell line was obtained using CRISPR/Cas9 system. MCF7 p53wt cells were stably transduced with lentivirus encoding pCW-Cas9 (#50661, Addgene) under doxycycline-inducible promoter, and then transfected with plasmid encoding *TP53*-specific or scrambled sgRNA using Gene Pulser Xcell Electroporation System (Bio-Rad, USA). Twenty-four hour prior electroporation 1 μg/ml doxycycline was added to cell medium. At second passage after electroporation monoclonal cell populations were obtained by fluorescence-activated cell sorting using BD FACSAria III (BD Biosciences, USA). *TP53* gene region in each population was DNA sequenced to confirm frameshift in proximity of Cas9-induced double-strand break. Western blot analysis was performed to validate lack of p53 protein.

sgRNA for *TP53* knockout (5′-GGTGCCCTATGAGCCGCCTG-3′) was cloned into pLenti-SG1, kindly provided by Prof. Ramziya Kiyamova, Kazan Federal University. 5′-CACCGGTGCCCTATGAGCCGCCTG-3′ and 5′-AAACCAGGCGGCTCATAGGGCACC-3′ oligonucleotides (EuroGene Ltd., Russia) were annealed to each other and cloned into vector via Esp3I restriction sites. Similarly, scrambled RNA (5′-GCACTACCAGAGCTAACTCA-3′) was cloned using 5′-CACCGCACTACCAGAGCTAACTCA-3′ and 5′-AAACTGAGTTAGCTCTGGTAGTGC-3′ oligonucleotides.

### Cell culture

HCT116 colorectal adenocarcinoma cells (ATCC/LGC Standards, UK), MCF7 p53wt and MCF7 p53^−/−^ (this study) human breast adenocarcinoma cell line were cultured under standard conditions in RPMI-1640 medium (PanEco, Russia) supplemented with 10% fetal bovine serum (Biosera, France), penicillin/streptomycin and 1mM l-glutamine. The cells were grown at 37 °C in an atmosphere of 5% CO_2_ in air.

### Treatments

Totally, 5 mM stock solutions of Complex and Ligand were prepared just before the experiments by dissolving the lyophilized compounds in DMSO. Cell treatments were performed using Complex at 50 μM, Ligand at 50 μM and 100 μM. For vehicle control equal volumes of 1% DMSO were added to untreated cells. Doxorubicin was used as a positive control at 4 μM.

### Immunoblot analysis

Cells were seeded in six-well plates at 5 × 10^5^ cells per well and cultured for 2 days followed by treatment with Complex (50 µM), Ligand (50 µM), DMSO (1%) for 24 h. Treated cells were harvested and lysed in RIPA buffer (Thermo Fischer Scientific, USA) containing 1 μl/ml Halt Protease and Phosphatase Inhibitor Cocktail with EDTA (Thermo Fischer Scientific, USA) according to manufacturer’s protocol. Whole-cell extracts were analyzed using Pierce BCA Protein Assay Kit (Thermo Fischer Scientific, USA) to determine total protein concentration. Samples were fractioned by 10% sodium dodecyl sulfate polyacrylamide gel electrophoresis and transferred to Immun-Blot polyvinylidene difluoride membrane using Trans-Blot SD Semi-Dry Transfer Cell (Bio-Rad, USA). Membranes were blocked with phosphate-buffered saline (PBS) with tween 20 containing 5% (mass/vol) nonfat dried milk for 1 h at RT, incubated with primary anti-p53 antibodies (Abcam, USA) overnight at 4 °C, and then with Anti-Mouse IgG–Peroxidase antibody (Sigma-Aldrich, USA) for 1 h. THE Beta Actin Antibody [HRP] (GenScript, USA) was used for detection of Beta Actin as loading control. Blots were developed with Clarity Western ECL Substrate (Bio-Rad, USA) and documented using ChemiDoc XRS Plus (Bio-Rad, USA).

### MTS assay

Cell viability was evaluated using colorimetric MTS assay that measures cellular metabolic activity. Cells were seeded into 96-well plates at 5 × 10^3^ cells per well in 80 μl full medium for 24 h, after that Complex (50 µM), Ligand (50 µM and 100 µM), DMSO (1%) and doxorubicin (4 μM) in 20 μl medium were added to appropriate wells and incubation continued for another 24 h. Then 20 μl of solution containing 2 mg/ml of MTS reagent (Promega, USA) and 150 μM phenazine methosulfate (Dia-M, Russia) was added to each well for 3 h at 37°С. The light absorbance at 490 nm was measured using Infinite M200 microplate reader (Tecan, Switzerland).

### Real-time cell proliferation assay

The experiments were performed using xCELLigence biosensor cell analysis system (ACEA Biosciences, USA). For real-time monitoring of cell proliferation 5 × 10^3^ cells were seeded in each well of E-plate 16 (ACEA Biosciences, USA) in full medium for 24 h. After that Complex (50 µM), Ligand (50 µM and 100 µM), DMSO (1%), and doxorubicin (4 μM) were added to appropriate wells and incubation continued for another 72 h. Cell index was registered every 15 min.

### Quantitative Taqman RT-PCR analysis

The standard procedure of total RNA extraction was performed using TRIzol Reagent (Thermo Fisher Scientific, USA) according to manufacturer’s protocol. Concentration of extracted RNA was determined by optical density measurement (A260/A280 ratio) using NanoDrop 2000 spectrophotometer (Thermo Fisher Scientific, USA). The reverse transcription reaction with 3 μg of total RNA was performed using 5× Reaction Buffer (Thermo Fisher Scientific, USA), RiboLock RNAse inhibitor (Thermo Fisher Scientific, USA), RevertAid (Thermo Fisher Scientific, USA), dNTP (Lytech, Russia) and random hexamer primer (Lytech, Russia). The reaction tubes were incubated at 25 °C for 10 min, at 42 °C for 60 min and at 70 °C for 10 min using С1000 Thermal Cycler (Bio-Rad, USA). Quantitative real-time PCR was carried out with 2.5× Master Mix (Sintol, Russia) using CFX96 Touch real-time PCR detection system (Bio-Rad, USA). Total volume of amplification reactions was 10 μl and each well contained 4 μl of 2.5× Master Mix, 1 μl of cDNA, 70-100 nM of both forward and reverse primers. Relative normalized expression was calculated by normalization to *Beta Actin*, data analyzed in CFX Manager software. All PCR reactions were performed in triplicates.

Quantitative Taqman RT-PCR was performed using the following primers and probes. *MDM2*: TGTGCAAAGAAGCTAAAGAAAAGG (fwd), AGGTTGTCTAAATTCCTAGGGTTAT (rev), [HEX]ATTGGTTGTCTACATACTGGGCAGGG[BHQ2] (probe). *p21/CDKN1A*: GCCTCCTCATCCCGTGTTCT (fwd), GTACCACCCAGCGGACAAGT (rev), [HEX]AGCCGGCCCACCCAACCTCCG[BHQ2] (probe). *PUMA*: GGGCCCGTGAAGAGCAAATG (fwd), CTGGCTCAGGGAAGATGGCT (rev), [FAM]CGGTTGCTCCAGCCCGGCGC[BHQ1] (probe). *Beta Actin*: GCGAGAAGATGACCCAGGATC (fwd), CCAGTGGTACGGCCAGAGG (rev), [HEX]CCAGCCATGTACGTTGCTATCCAGGC[BH2] (probe).

### Flow cytometry analysis

Quantification of apoptotic cells was performed for MCF7 p53wt and MCF7 p53^−/−^ cells treated with Complex (50 µM), Ligand (50 µM), and DMSO (1%) for 48 h. Treated cells were harvested by trypsinization, washed with Dulbecco's PBS and stained using APC Annexin V Apoptosis Detection Kit with Propidium Iodide (Sony Biotechnology, USA) according to manufacturer’s protocol. Stained cells were immediately analyzed by flow cytometry using BD FACSAria III (BD Biosciences, USA) and data processed with FlowJo software package (FlowJo LLC, USA).
